# Altered nasal and oral microbiomes define pediatric sickle cell disease

**DOI:** 10.1128/msphere.00137-25

**Published:** 2025-05-14

**Authors:** Audra L. Crouch, Beatrice M. Severance, Susan Creary, Darryl Hood, Michael Bailey, Asuncion Mejias, Octavio Ramilo, Michelle Gillespie, Stefanie Ebelt, Vivien Sheehan, Benjamin T. Kopp, Matthew Z. Anderson

**Affiliations:** 1 Department of Microbiology, The Ohio State University, Columbus, Ohio, USA; 2 Laboratory of Genetics, University of Wisconsin—Madison, Madison, Wisconsin, USA; 3 Research Institute at Nationwide Children’s Hospital, Columbus, Ohio, USA; 4 College of Public Health, The Ohio State University, Columbus, Ohio, USA; 5 Department of Infectious Diseases, St Jude Children’s Research Hospital, Memphis, Tennessee, USA; 6 Environmental Health and Epidemiology, Rollins School of Public Health at Emory University, Atlanta, Georgia, USA; 7 Children’s Healthcare of Atlanta, Atlanta, Georgia, USA; 8 Division of Pulmonology, Asthma, Cystic Fibrosis, and Sleep, Emory University School of Medicine, Atlanta, Georgia, USA; 9 Department of Microbial Infection and Immunity, The Ohio State University, Columbus, Ohio, USA; 10 Center for Genomic Science Innovation, University of Wisconsin—Madison, Madison, Wisconsin, USA; University of Michigan Medical School, Ann Arbor, Michigan, USA

**Keywords:** sickle cell disease, oral microbiome, nasal microbiome

## Abstract

Sickle cell disease (SCD) is a chronic blood disorder that disrupts multiple organ systems and can lead to severe morbidity. Persistent and acute symptoms caused by immune system dysregulation in individuals with SCD could contribute to disease either directly or indirectly via dysbiosis of commensal microbes and increased susceptibility to infection. Here, we explored the nasal and oral microbiomes of children with SCD (cwSCD) to uncover potential dysbiotic associations with the blood disorder. Microbiota collected from nasal and oral swabs of 40 cwSCD were compared to eight healthy siblings using shotgun metagenomic sequencing. Commensal taxa were present at similar levels in the nasal and oral microbiome of both groups. However, the nasal microbiomes of cwSCD contained a higher prevalence of Pseudomonadota species, including pathobionts such as *Yersinia enterocolitica* and *Klebsiella pneumoniae*. Furthermore, the oral microbiome of cwSCD displayed lower α-diversity and fewer commensal and pathobiont species compared to the healthy siblings. Thus, subtle but notable shifts seem to exist in the nasal and oral microbiomes of cwSCD, suggesting an interaction between SCD and the microbiome that may influence health outcomes.

**IMPORTANCE:**

The oral and nasal cavities are susceptible to environmental exposures including pathogenic microbes. In individuals with systemic disorders, antibiotic exposure, changes to the immune system, or changes to organ function could influence the composition of the microbes at these sites and the overall health of individuals. Children with sickle cell disease (SCD) commonly experience respiratory infections, such as pneumonia or sinusitis, and may have increased susceptibility to infection because of disrupted microbiota at these body sites. We found that children with SCD (cwSCD) had more pathobiont bacteria in the nasal cavity and reduced bacterial diversity in the oral cavity compared to their healthy siblings. Defining when, why, and how these changes occur in cwSCD could help identify specific microbial signatures associated with susceptibility to infection or adverse outcomes, providing insights into personalized treatment strategies and preventive measures.

## INTRODUCTION

Sickle cell disease (SCD) is a chronic blood disorder that encompasses an assemblage of genetically inherited blood disorders such as sickle cell anemia (SCA), hemoglobin sickle C disease, and β-thalassemia (1, 2). The sickle-shaped red blood cell stems from the inheritance of two beta hemoglobin gene mutations that produce hemoglobin prone to polymerization in deoxygenated environments (3). SCD leads to vaso-occlusive events that can cause pain and stroke and end with organ damage and also acute chest syndrome (ACS) episodes, which are instigated by pulmonary embolisms, pulmonary infarctions, pulmonary fat embolisms, and infections (2, 4–9). Together, these systemic complications have lasting impacts on quality of life, neural and organ function, and immune defense in people with SCD and increase the risk of mortality (2, 3).

The nasal and oral cavities serve as the frontline defense against foreign particulates and pathogenic microbes from the environment (10, 11). In healthy individuals, the nasal passage is initially colonized by *Staphylococcus* after birth, and the nasal microbial diversity increases by replacement of *Staphylococcus* with *Corynebacterium*, *Cutibacterium*, *Dolosigranulum*, *Streptococcus*, and Enterobacteriaceae species as the child develops (10, 12, 13). In contrast, the oral cavity is first colonized by *Streptococcus* but rapidly diversifies its taxonomic profile to include *Neisseria*, *Haemophilus*, *Porphyromonas*, and multiple *Streptococcus* species by 1 year of age (11, 14, 15).

The role of the microbiome in SCD and its potential impact on health through childhood development and disease is less clear. Most research investigating microbial associations with SCD has focused on respiratory and systemic pneumococcal infections and other outcomes resulting from immune system dysfunction (4, 16–19). However, the gut microbiome of adults with SCD is known to display shifts in taxonomic abundance. Candidatus Saccharibacteria, Bifidobacteriaceae, Clostridiales, and Fusobacteriaceae genera are more prevalent in the gut of individuals with SCD, and Pasteurellaceae, Bacillaceae, Prevotellaceae, and Verrucomicrobiaceae species are less abundant compared to those without SCD (20). Furthermore, when stool from mice with SCD was transplanted into healthy mice, the SCD gut contents induced persistent pain in the recipient mice. Pain relief in mice with transplanted SCD microbiota could be conferred by probiotic treatment with *Akkermansia mucinophila* (21). Collectively, these studies suggest that the gut microbiome is influenced by SCD and may impact symptom severity. In contrast, the impact of SCD on the basal upper airway microbiome has only been described in a limited fashion (22), although symptoms associated with SCD and the corresponding treatment regimens modulate oral and nasal microbiome diversity and succession in individuals without SCD (23–28).

Here, we taxonomically profiled the nasal and oral microbiomes of children with SCD (cwSCD) and their healthy siblings as part of the Sickle Cell Disease Microbiologic and Immunologic Links to Health Equity study. Microbial diversity in the nasal cavity was similar between the two groups, with the exception of a greater abundance of Pseudomonadota in the nasal cavity of cwSCD. In contrast, α-diversity of the oral microbiome was lower in the SCD cohort compared to the healthy controls. cwSCD had a reduced representation of the 20 most abundant commensal and pathobiont species in the oral cavity when compared to the healthy population. Overall, this work identified that there may be subtle shifts in the nasal and oral microbiomes of cwSCD that indicate dysbiotic conditions that are linked to poor health outcomes.

## RESULTS

One nasopharyngeal and one oropharyngeal swab were collected from 40 cwSCD <18 years. Healthy siblings of participants served as healthy controls (HC; *N* = 8) for environmental and genetic influences on the associated host microbiome. Table 1 shows participant demographics and disease status, including average age, biological sex, and the frequency of asthma, allergic rhinitis, prior ACS events, prior hydroxyurea treatment, prior antibiotic treatment, and hospitalizations for the cwSCD.

**TABLE 1:** Demographics and medical history of the cwSCD and HC cohorts^*a*^

Parameter	SCD subjects, *N* = 40 (%)	HC subjects, *N* = 8
Age	8.1 ± 5.3	11 ± 4.0
Sex (male/female/undefined)	21/18/1	4/4
Allergic rhinitis	7 (17.5)	n/a^*b*^
Asthma	10 (25)	n/a
ACS	14 (40)	n/a
Hydroxyurea	26 (65)	n/a
Antibiotic usage	19 (47.5)	n/a
Hospitalizations	10 (25)	n/a

^*a*^ All data collected for medical treatments, hospitalizations, and chronic conditions were reported from the previous year.

^*b*^ "n/a,” not applicable.

To explore the impact of SCD on the nasal and oral microbiomes, we extracted total genomic DNA from each oral and nasal swab and selectively removed the human-derived nucleic acids. Depletion of human material led to the removal of multiple samples due to low remaining microbial DNA, leaving only 24 cwSCD and 4 HC nasal samples and 38 cwSCD and 7 HC oral samples for sequencing (Fig. S1). We performed shotgun metagenomic sequencing on the remaining samples to a depth of 40 million reads per sample. Sequenced nasal samples had more residual contaminating human DNA than oral samples (e.g., 66.3% vs 31.7% on average, respectively), and samples from cwSCD had higher amounts of human material than the sibling controls (77.01% vs 45.96% in the nasal samples and 35.36% vs 31.07% in the oral samples, respectively; Fig. S 2A). The high level of human contamination in one nasal cwSCD sample resulted in its removal from further analysis due to low remaining microbial read counts. After human DNA removal and quality control processing, all remaining samples had a minimum of 5.45 log_10_ reads. Oral microbiome samples from both groups had higher read numbers (median of 7.27 and 7.15 log_10_ reads for cwSCD and HC, respectively) compared to nasal samples that had median values of 6.46 and 6.78 log_10_ reads for cwSCD and HC individuals, respectively (Fig. S2B).

### Oral microbiomes were distinct from nasal microbiomes in children with SCD

To define the microbial composition of each oral and nasal sample, reads were taxonomically assigned using Kraken2 (Fig. S1). Notably, nasal swabs contained a greater proportion of taxonomically assigned reads compared to oral swabs; 90.06% and 89.46% of metagenomic reads from cwSCD and HC nasal samples were taxonomically assigned, respectively, vs only 75.41% and 67.34% of reads from cwSCD and HC oral samples, respectively (Fig. S3). However, there was no statistical difference in the relative abundance of read assignment between cwSCD and HC in either nasal or oral samples.

**Fig 1:**
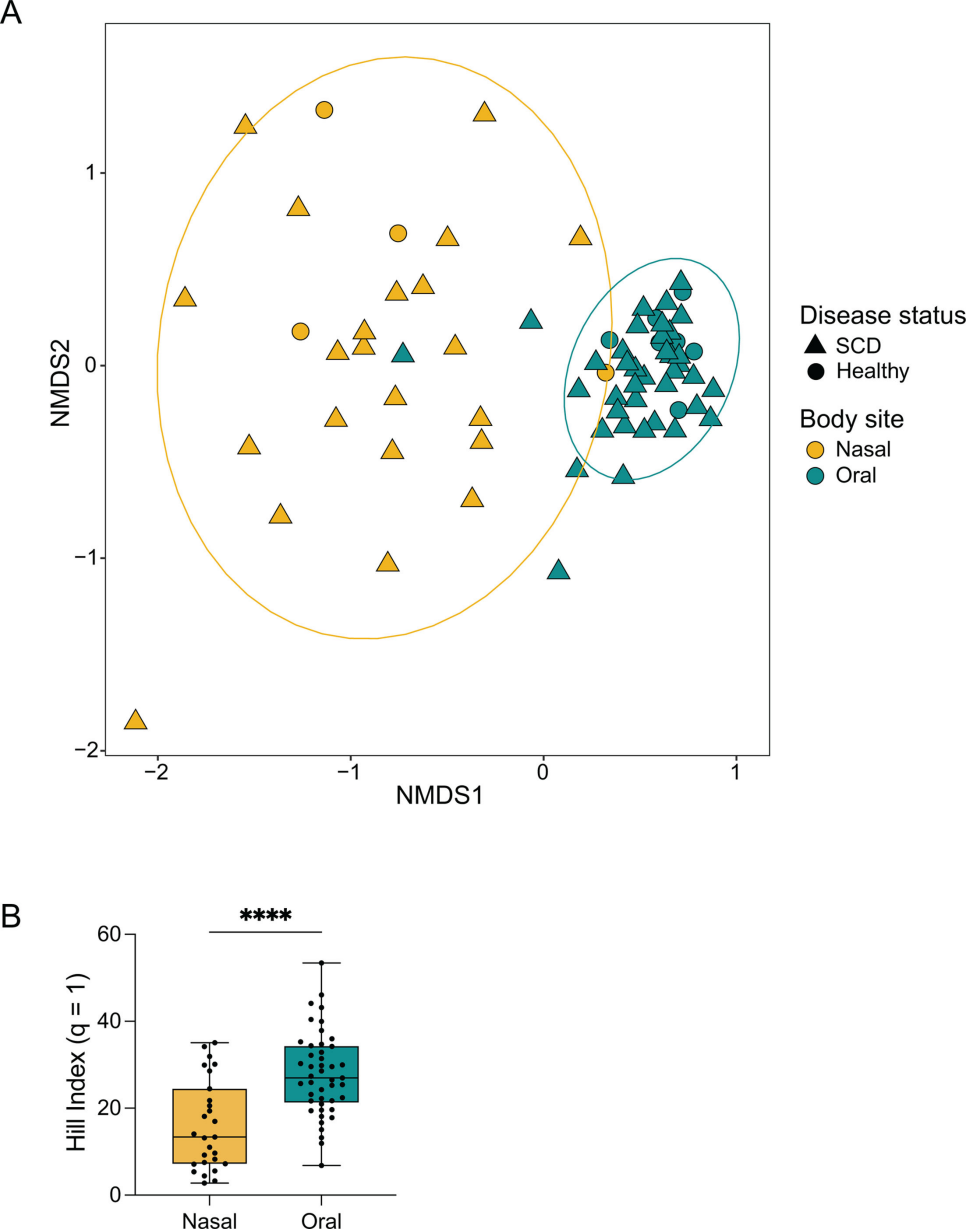
The nasal and oral microbiome are distinct in pediatric SCD and healthy individuals. (A) Bray-Curtis dissimilarity index is visualized for β-diversity with all nasal and oral samples using NMDS ordination. (**B**) Hill (*q* = 1) α-diversity index is plotted for each nasal and oral sample. Mann-Whitney *U* test: ****, *P*-value < 0.0001. *N* = 27 for nasal samples and *N* = 45 for oral samples. Goldenrod represents nasal samples, and teal represents oral samples.

To determine if compositional differences existed between the nasal and oral microbiomes of cwSCD and HC individuals, we visualized sample clustering by Bray-Curtis dissimilarity with non-metric multidimensional scaling (NMDS) distance ordination. Nasal and oral microbiomes clustered separately with minimal overlap (Fig. 1A; permutational analysis of variance [PERMANOVA], *r*^2^ = 0.263, *P* = 9.90 × 10^−4^). Oral samples possessed higher mean Hill (*q* = 1) α-diversity scores with a median of 27.0 compared to a nasal mean Hill median value of 13.4 (Fig. 1B; Mann-Whitney *U* test, *U* = 254, *P*-value < 0.0001). In contrast, the microbiome of cwSCD and HC samples clustered together for each host niche. Together, these data indicate significant taxonomic differences between oral and nasal samples but comparatively little influence of SCD on large-scale microbial community composition.

### Pathogenic species are more prominent in the nasal microbiomes of cwSCD

**Fig 2:**
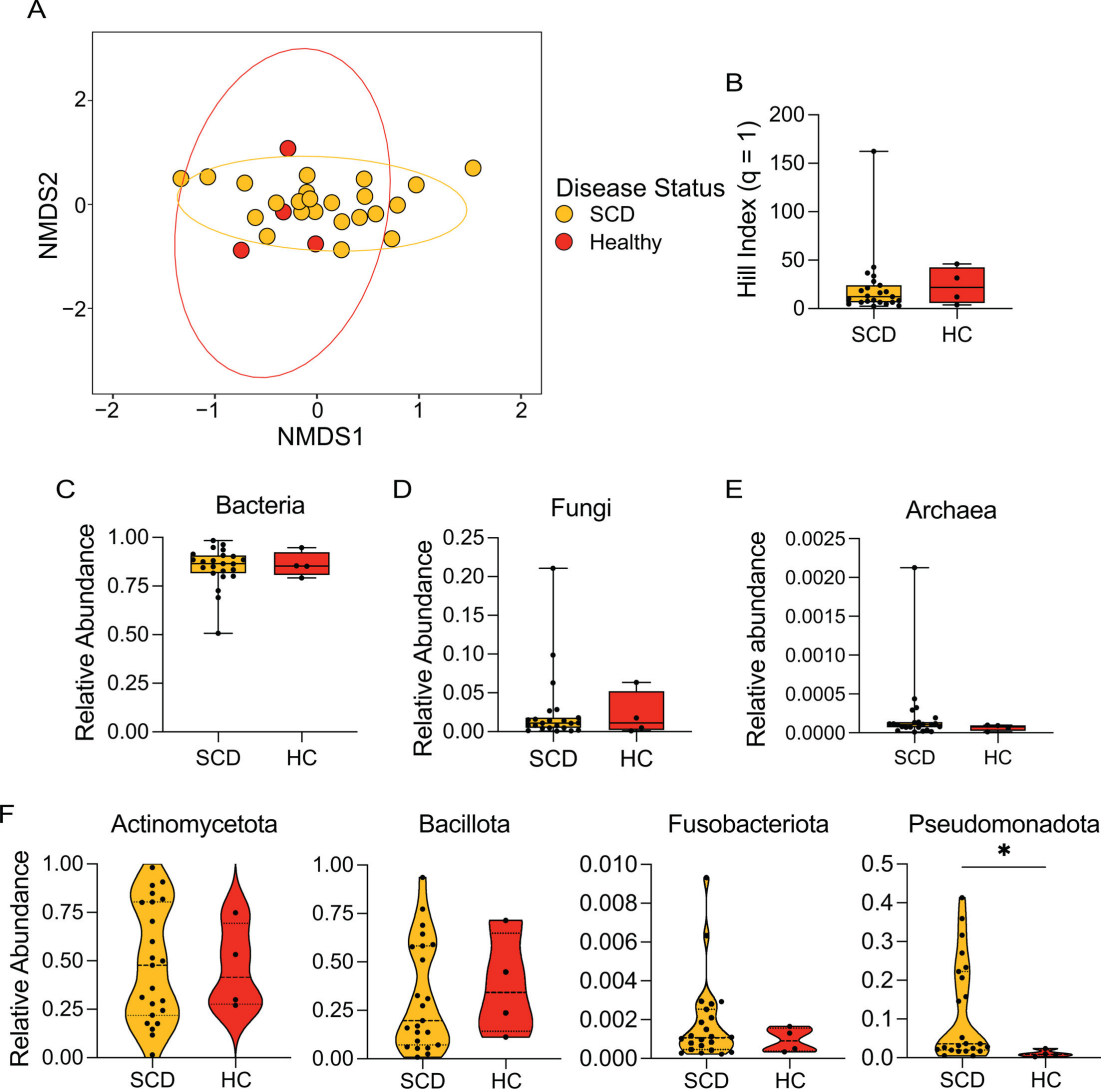
cwSCD nasal samples have an increased representation of Pseudomonadota compared to healthy controls. (A) Nasal samples were visualized using the Bray-Curtis dissimilarity index for β-diversity for cwSCD and healthy control subjects. (**B**) Hill (*q* = 1) α-diversity index is plotted for each cwSCD and HC subject. The relative abundance for Bacteria (**C**), Fungi (**D**), Archaea (**E**), and major bacterial phyla (**F**) for each cwSCD and HC microbiome sample is given. *N* = 23 for cwSCD and *N* = 4 for HC. Mann-Whitney *U* test: *, *P*-value < 0.05. Goldenrod represents cwSCD samples, and red represents HC samples.

To identify if the major taxonomic differences between host sites obscured microbial signatures of cwSCD, we separated the microbiota profiles of each host niche. In the nasal samples, clustering indicated little difference between the microbial community structure of cwSCD and HC samples (Fig. 2A; PERMANOVA, *r*^2^ = 0.0448, *P* = 0.293). This is further supported by the lack of statistical difference in the α-diversity Hill metric (*q* = 1) between cwSCD and HC individuals (Fig. 2B; Mann-Whitney *U* test, *U* = 38, *P*-value = 0.6217).

The nasal microbiome in both cwSCD and HC children was primarily composed of bacteria, accounting for 86.51% and 85.29% of reads, respectively (Fig. 2C; Mann-Whitney *U* test, *U* = 43, *P*-value = 0.869). Fungi constituted a much smaller but noticeably present proportion of the community, with median relative abundances of 1.10% vs 1.13% for cwSCD and HC children, respectively (Fig. 2D; Mann-Whitney *U* test, *U* = 45.0, *P*-value = 0.974). Finally, archaea represented the smallest portion of taxonomic reads for both cwSCD and HC subjects (0.0108% vs 0.00775%; Fig. 2E; Mann-Whitney *U* test, *U* = 26.0, *P*-value = 0.191). Together, these data indicate little kingdom-level variation between the nasal microbiomes of cwSCD and HC individuals.

Differences in the nasal microbiome of cwSCD and HC individuals could be detected at the phylum level. Pseudomonadota had a 4.15× higher relative abundance in the cwSCD microbiome compared to HC children (0.036 vs 0.0087 relative abundance, respectively; Fig. 2F; Mann-Whitney *U* test, *U* = 11, *P*-value = 0.0138). The other major bacterial phyla were similar between the cwSCD and HC groups: Actinomycetota (Mann-Whitney *U* test, 0.477 vs 0.416, *U* = 46.0, *P*-value = 1), Bacillota (0.197 vs 0.343, *U* = 37.0, *P*-value = 0.576), and Fusobacteriota (0.00109 vs 0.000906, *U* = 39.0, *P*-value = 0.669).

**Fig 3:**
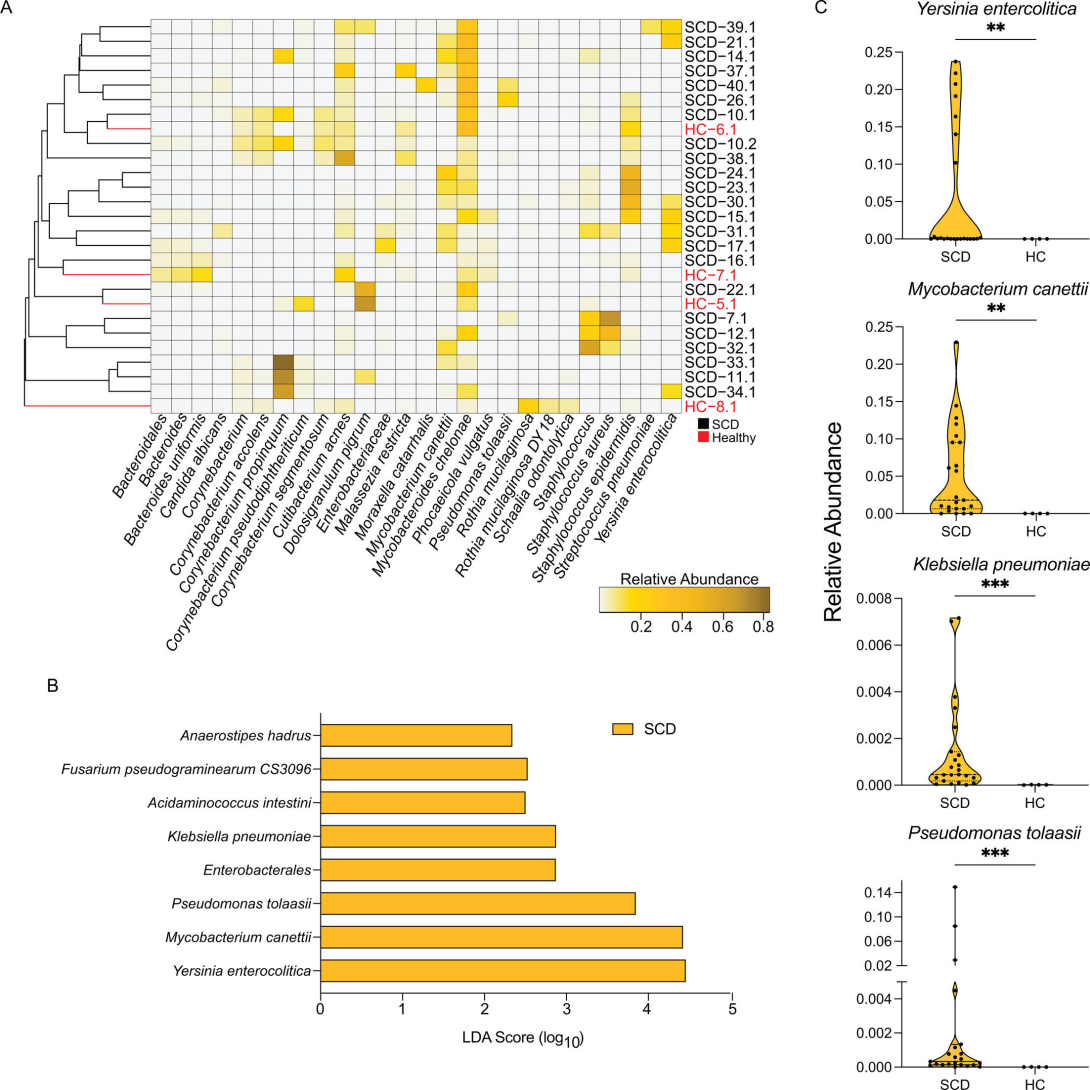
cwSCD nasal samples have an increased representation of specific pathobionts compared to healthy controls. (A) Twenty-six species were identified with at least 5% representation in one cwSCD sample. The relative abundance of these species in cwSCD and HC samples was plotted as a heatmap. Average linkage clustering was applied to a Bray-Curtis dissimilarity matrix to display sample similarity. Black text represents cwSCD, and red text represents HC subjects. (**B** ) Linear discriminate analysis effect size (LEfSe) analysis determined differentially abundant taxa between cwSCD and the HC cohorts. (**C)**) The relative abundance of the three pathobiont species and one environmental taxon was detected by LEfSe analysis. *N* = 23 for SCD and *N* = 4 for HC. Mann-Whitney *U* test: ***, *P*-value < 0.001; **, *P*-value < 0.01. Goldenrod represents cwSCD samples, and red represents HC samples.

To uncover highly abundant microbial species that could define the sickle cell nasal microbiome, we identified taxa with a relative abundance of at least 0.05 in at least one cwSCD sample (Fig. 3A). cwSCD and HC microbiome profiles were intermingled when clustered based on taxa abundance (Fig. 3A), which is supported by their similarities in α- and β-diversity. Even among the more prevalent taxa in cwSCD samples, relatively few species distinguished the two groups. Two Pseudomonadota species, *Pseudomonas tolaassii* and *Yersinia enterocolitica*, were significantly more abundant in cwSCD samples and could be driving the increased Pseudomonadota representation in the nasal microbiota of cwSCD (Fig. 3C; Mann-Whitney *U* test, *P. tolaassii*, *U* = 1, *P*-value = 0.0002; *Y. enterocolitica*, *U* = 0.021, *P*-value = 0.0014). Remarkably, two fungal species, *Candida albicans* and *Malassezia restricta*, were found among the most abundant species in cwSCD (Fig. 3A). However, no statistical difference in abundance was present between cwSCD and HC samples for these two fungal species (Fig. S4A; Mann-Whitney: *C. albicans*, *U* = 32, *P*-value = 0.3715, *M. restricta*, *U* = 39, *P*-value = 0.6688). Therefore, their inclusion among the most abundant taxa in cwSCD was likely a consequence of a few samples with extremely high proportions of fungi.

To identify all taxa differences between the cwSCD and HC cohorts in the nasal cavity, we conducted a systematic comparison of taxonomical differential abundance. Eight taxa differentiated the cwSCD and HC individuals by linear discriminate analysis effect size (LEfSe) (29). All eight species exhibited higher prevalence in cwSCD and included the two abundant Pseudomonadota species described above (Fig. 3B). Five of the eight species were common human-associated commensal species or could be commonly encountered in the environment, including the fungal species *Fusarium pseudograminearum*, a common grain pathogen (30). LEfSe analysis detected notable differences for three pathogenic or pathobiont species. Notably, the common gastrointestinal pathogen *Y. enterocolitica* displayed a 176.5× higher relative abundance in cwSCD compared to the healthy sibling cohort (median relative abundance of 4.77 × 10^−4^ and 2.71 × 10^−6^, respectively; Fig. 3C; linear discriminate analysis [LDA] = 4.46). The abundance of *Y. enterocolitica* in some cwSCD exceeded 20% of total reads, suggestive of extreme dysbiosis. Similarly, *Mycobacterium canettii* (0.018 vs 4.38 × 10^−6^, LDA = 4.43) and *Klebsiella pneumoniae* (0.00456 vs 1.83 × 10^−5^, LDA = 2.88) were 4,118× and 2.5× more abundant in cwSCD nasal microbiomes compared to HC, respectively (Fig. 3C; Mann-Whitney *U* test: *M. canettii U* = 4, *P*-value = 0.0014; *K. pneumoniae U =* 2, *P*-value = 5.00 × 10^−4^). Both *M. canettii* and *K. pneumoniae* are common respiratory pathogens that pose a significant health risk for individuals with compromised immune systems or lung function (19, 31). Thus, cwSCD carry higher loads of particular pathobionts that may predispose them toward infections.

We hypothesized that cwSCD may have a higher carrying capacity of pathobiont or pathogenic species in the nasal cavity. To address this, we screened the identified taxa in the nasal microbiomes and isolated abundance data for known pathogens and pathobionts of the airways and nasal nares. Comparison of overall pathogen and pathobiont profiles found a 3.2× higher relative abundance in cwSCD compared to their HC siblings but failed to reach statistical significance (Fig. S5; Mann-Whitney *U* test, *U* = 19, *P*-value = 0.0694). Overall, these data suggest that cwSCD harbor a higher prevalence of specific pathogens and pathobionts that may be true more broadly in the nasal cavity.

### cwSCD have reduced microbial diversity in the oral cavity

**Fig 4:**
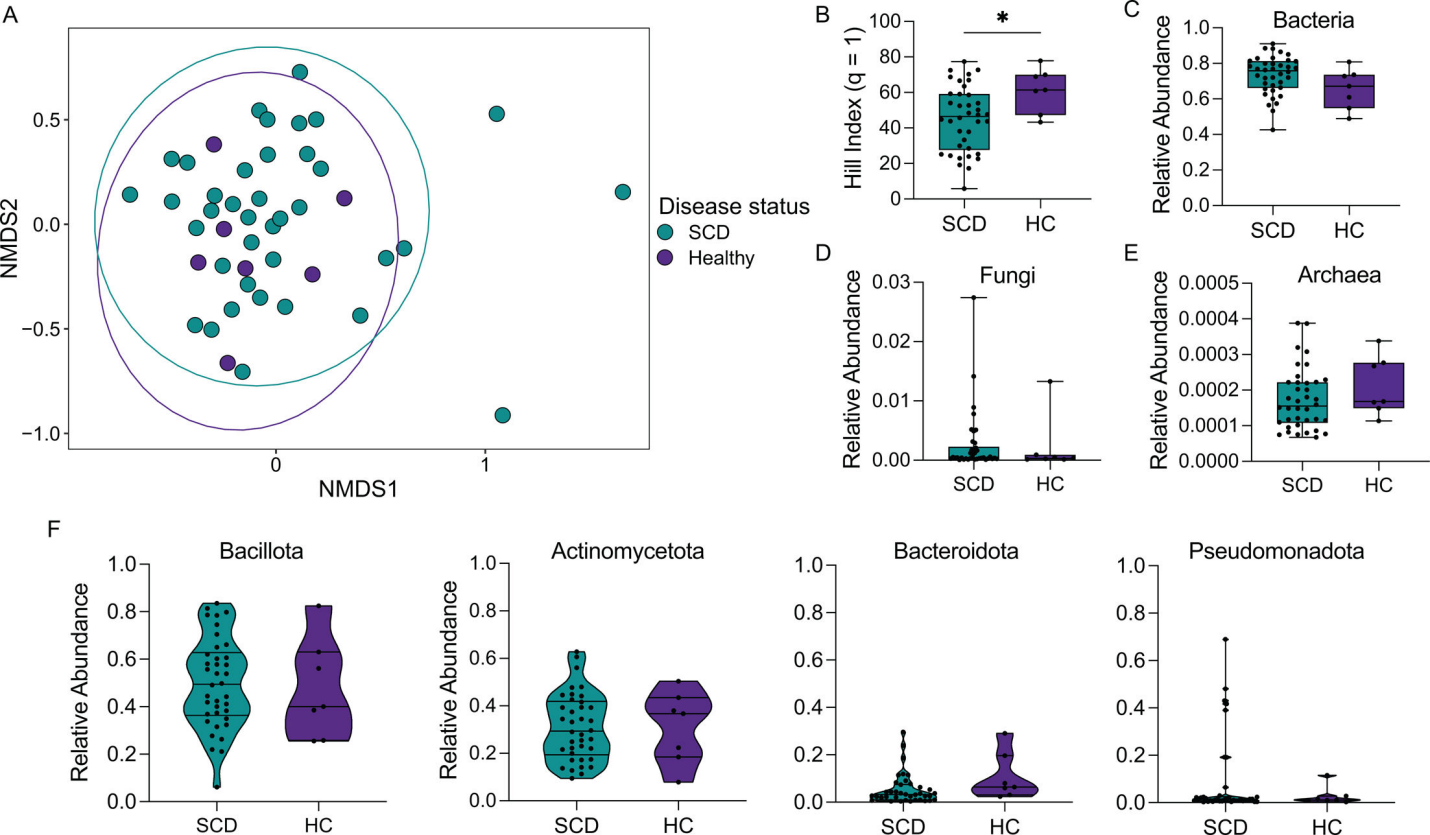
cwSCD oral samples have reduced *α*-diversity compared to healthy controls. (A) Oral samples w**e**re visualized using the Bray-Curtis dissimilarity index for β-diversity for cwSCD and HC cohorts. (**B**) Hill (*q* = 1) α-diversity index is plotted for each cwSCD and HC subjects. The relative abundance for bacteria (**C**), fungi (**D**), archaea (**E**), and major bacterial phyla (**F**) for each cwSCD and HC individual is given. *N* = 38 for SCD and *N* = 7 for HC. Mann-Whitney *t*-test: *, *P*-value < 0.05. Teal represents cwSCD samples, and purple represents HC samples.

We next explored the taxonomic signatures of oral microbiomes in the cwSCD and HC pediatric cohorts. NMDS ordination of a Bray-Curtis dissimilarity index indicated there was no difference between the oral microbiomes of cwSCD or their HC siblings (Fig. 4A; PERMANOVA, *r*^2^ = 0.02345, *P*-value = 0.393). HC individuals had increased α-diversity by the Hill metric (*q* = 1), with a mean of 61.4 compared to the cwSCD mean of 45.2 (Fig. 4B; Mann-Whitney *U* test, *U* = 67.0, *P*-value = 0.0383), indicating increased evenness and richness.

The oral microbiomes of cwSCD and HC participants were dominated by bacteria (Fig. 4C). We found that cwSCD had a higher abundance of bacteria, though statistically insignificant, than HC subjects with median abundances of 73.3% and 65.6%, respectively (Fig. 4C; Mann-Whitney *U* test, *U* = 79.0, *P*-value = 0.0944). Fungi were substantially less abundant and had similar median relative abundances of 0.0460% and 0.0325% for cwSCD and HC subjects, respectively (Fig. 4D; Mann-Whitney *U* test, *U* = 104, *P*-value = 0.380). Finally, archaea were the least abundant taxa with median values of 0.0155% vs 0.0168% in cwSCD and HC subjects, respectively (Fig. 4E; Mann-Whitney *U* test, *U* = 96.0, *P*-value = 0.259). Thus, similar to the nasal microbiomes, little variation existed between the cwSCD cohort and the healthy cohort across microbial kingdoms.

There was no statistically significant variation between the cwSCD and HC cohorts among bacterial phyla. The most abundant phyla present in the cwSCD and healthy siblings were Bacillota (Fig. 4F; Mann-Whitney *U* test, 0.495 vs 0.401, *U* = 120, *P*-value = 0.702), Actinomycetota (0.294 vs 0.367, *U* = 130, *P*-value = 0.939), Bacteroidota (0.0321 vs 0.0638, *U* = 76.0, *P*-value = 0.0765), and then Pseudomonadota (0.0151 vs 0.0113, *U* = 126, *P*-value = 0.842).

**Fig 5:**
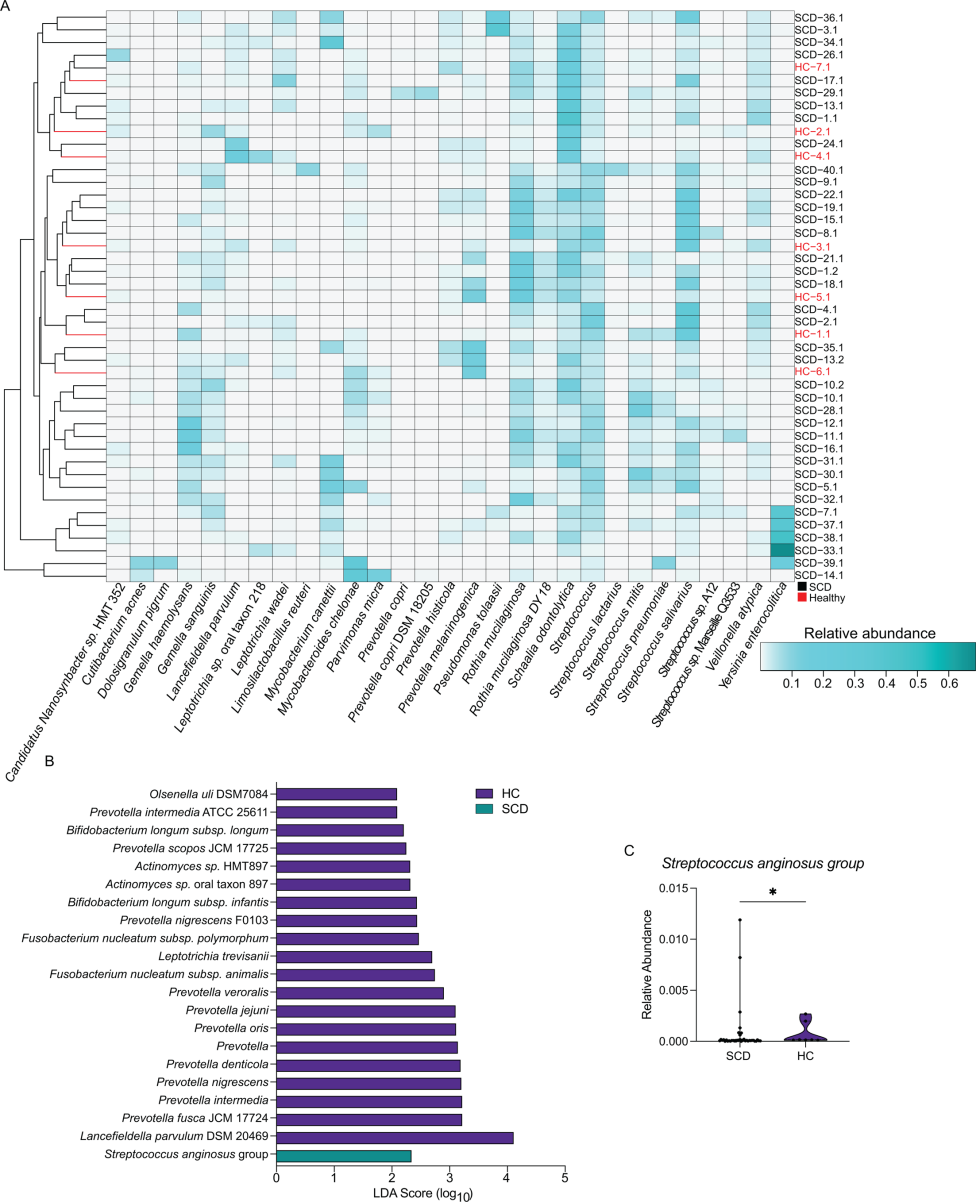
cwSCD oral samples have a reduction of commensal and pathobiont species compared to healthy subjects. (A) Twenty-nine species were identified with at least 5% representation in one cwSCD sample. The relative abundance in cwSCD and HC samples was plotted as a heatmap. Average linkage clustering was applied to a Bray-Curtis dissimilarity matrix to display sample similarity. Black text represents cwSCD, and red text represents HC individuals. (**B**) LEfSe analysis determined differentially abundant taxa between cwSCD and the HC cohorts. (**C**) The relative abundance of the single pathobiont group was detected by LEfSe analysis. *N* = 38 for SCD and *N* = 7 for HC. Mann-Whitney *t*-test: **, *P*-value < 0.01 ; ***, *P*-value < 0.001. Teal represents cwSCD samples, and purple represents HC samples.

To determine if the most prevalent species in the oral microbiomes of cwSCD can distinguish them from the HC cohort, we assessed individual taxa with a relative abundance of at least 0.05 in at least one subject (Fig. 5A). As observed for nasal microbiomes, the dendrogram built from a Bray-Curtis dissimilarity matrix intermixed cwSCD and HC individuals, indicating little difference between the two groups (Fig. 5A). Among the 20 most abundant taxa in the cwSCD oral niche, no individual species was significantly different between the cwSCD and HC cohort. Three fungal taxa were consistently present in the oral cavity but were not statistically different between groups (Fig. S4B): *C. albicans* (Mann-Whitney *U* test, 1.82 × 10^−4^ vs 1.73 × 10^−4^, *U* = 126, *P*-value = 0.765), *Fusarium pseudoperiodonticum* (3.46 × 10^−4^ vs 5.02 × 10^−4^, *U* = 86.0, *P*-value = 0.128), and *M. restricta* (5.96 × 10^−5^ vs 1.08 × 10^−4^, *U* = 112, *P*-value = 0.471).

We identified 21 taxa through LEfSe analysis that distinguished SCD and HC children within the oral cavity (Fig. 5B). Twenty of these species were statistically more prevalent in the oral microbiome of the control siblings compared to cwSCD, including *Prevotella spps.* and *Actinomyces spps.*, all of which were a mix of commensal and pathobiont species (Fig. 5B). Notably, this included two pathobiont *Fusobacterium nucleatum* subspecies that are known for their associations with periodontitis, oral abscesses, and multiple cancers (32–34). A single taxon, *Streptococcus anginosus* group, was statistically overrepresented in the cwSCD oral microbiome. *S. anginosus* is a common group of oral pathobiont species that can cause infections at multiple other resident body sites (35). Although LEfSe considered *S. anginosus* to be more prevalent in cwSCD, the healthy control cohort had a higher median value than cwSCD (1.48 × 10^−4^ vs 8.22 × 10^−5^, respectively; Fig. 5C; LDA = 2.34, Mann-Whitney *U* test, *U* = 66.0, *P*-value = 0.0352). A more focused analysis of pathogen and pathobiont profiles in the oral cavity found no significant difference between the two subject groups (Fig. S5C; PERMANOVA, *r*^2^ = 0.0298, *P* = 0.208). Furthermore, statistical analysis revealed no significant difference in the calculated ratio of pathogen-pathobionts to commensals (0.119 vs 0.107; Fig. 5C; Mann-Whitney *U* test, *U* = 132, *P*-value = 0.988). Taken together, cwSCD had reduced oral microbial diversity and significantly decreased the abundance of many *Prevotella* species.

### Prior medical history does not impact the cwSCD microbiome

The relatively small cohort included here is underpowered to be able to detect any but large contributors to microbiome variation between the cwSCD and HC cohorts. Nonetheless, demographic information and prior medical history obtained at enrollment had little impact on microbiota composition. Biological sex had no impact on nasal or oral community diversity between cwSCD and HC subjects (Table 2; PERMANOVA and Kruskal-Wallis test). Furthermore, no comorbidities or treatments were significantly associated with nasal or oral microbiome community structure and diversity for cwSCD, including asthma, allergic rhinitis, prior ACS, prior acute antibiotic treatment, or past hydroxyurea treatment (Table 2; PERMANOVA and Mann-Whitney *U* test).

**TABLE 2:** Statistical analysis of demographics and medical history of the cwSCD and HC cohorts

Parameter	Nasal samples			Oral samples		
	PERMANOVA *r*^2^	PERMANOVA *P*-value	α-Diversity *P*-value	PERMANOVA *r*^2^	PERMANOVA *P*-value	α-Diversity *P*-value
Biological sex	0.0841	0.326	0.148	0.0368	0.705	0.267
Asthma	0.0624	0.467	0.221	0.0593	0.067	0.355
Allergic rhinitis	0.0917	0.138	0.0780	0.0283	0.585	0.302
Antibiotic	0.0371	0.834	0.874	0.0260	0.685	0.503
Prior ACS	0.0611	0.0449	0.140	0.0482	0.147	0.287
Hydroxyurea	0.0315	0.917	0.746	0.0222	0.808	0.792

## DISCUSSION

Sickle cell disease is an understudied chronic blood disorder that can compromise the function of major organ systems in the human host. Previous SCD research has primarily focused on symptom management and gene therapy (36, 37), but studies have demonstrated gut microbiota shifts in individuals with SCD (20, 21, 38, 39). In this study, we expanded the analysis of the gut microbiome to the nasal and oral cavity in cwSCD at their baseline state of health. Subtle but significant differences in microbial diversity occurred within these niches for cwSCD compared to their healthy siblings. Overall, cwSCD had reduced microbial diversity in the oral cavity and an increased abundance of pathobionts in the nasal passage.

When comparing the oral and nasal microbiomes of children with or without SCD, we found strong differences between the two body sites. Furthermore, α-diversity was significantly greater in the oral cavity compared to the nasal cavity. These data are similar to results in other studies comparing the two body sites in both healthy and diseased subjects (11, 40–45).

The cwSCD nasal microbiome generally resembled the nasal microbiome of healthy individuals except for a few species. The major taxonomic feature that defined the cwSCD nasal microbiome was an increased prevalence of Pseudomonadota phyla, likely driven by the increased abundance of the two pathobiont species *Y. enterocolitica* and *K. pneumoniae* and the environmental species *P. tolaasii*. Increased prevalence of these two pathobiont species and the Actinomycetota lung pathogen *M. canettii* could indicate an increased risk of pathobiont carriage and an increased risk of infection in the upper airways of cwSCD due to a compromised immune system (2, 6, 7, 9, 36, 46, 47). However, SCD status had little effect on core commensal species such as *Corynebacterium spps.* or *Staphylococcus spps.* Thus, it is feasible that the nasal microbiome may play an integral role in the disease and respiratory health of individuals with SCD, although it is not possible to disentangle the cause and effect of this association here and would require testing directly in animal models of SCD seeded with species increased in the nasal cavity (48).

The oral microbiome of the cwSCD cohort had a reduction in alpha diversity when compared with healthy siblings. Reduced alpha diversity of the oral microbiome is similarly observed in other disease states, such as oral cancers and diabetes (49–51), but this reduced diversity may not reflect dysbiosis in the oral cavity of cwSCD. Eight of the 20 species or strains that were more prevalent in the oral microbiome of the HC cohort are associated with various oral disease states (32, 52–56), indicating that increased diversity may not always be an indicator of host health. However, neither cohort showed a significant difference in the pathobiont-to-commensal ratio. Interestingly, *Prevotella* species were less abundant in the cwSCD oral microbiome. Many of the identified taxa are considered standard commensals of the oral cavity and may indicate a loss of microbial diversity in cwSCD that could leave individuals vulnerable to pathobiont proliferation and disease (53). Understanding the microbiome variation in the oral cavity could have implications in targeted therapeutic strategies to manage dental disease, infections, and inflammatory conditions that are more common in individuals with SCD (57–60).

Interestingly, patient history had little impact on the microbiome of cwSCD. While a lack of association between biological sex and the microbiome was expected (14), we had anticipated that prior adverse events would put individuals at greater risk of microbial dysbiosis. These analyses were, however, significantly limited by our small sample size. Longitudinal profiling and a greater number of participants are needed to uncover the complex interactions between the nasal and oral microbiome’s taxonomic structure, acute events like ACS, and the influence of systemic drug therapies.

In conclusion, cwSCD seem to have subtle shifts in their nasal and oral microbiomes at baseline compared to healthy cohorts, implicating several avenues for understanding and managing the disease. Investigation of microbiome changes during acute events in cwSCD may provide more insight into the microbial drivers of adverse outcomes that are less clear when individuals are presenting at baseline. Nonetheless, this work begins to lay the foundation for the potential management of disease by understanding how microbial changes may contribute to disease and susceptibility to adverse outcomes. Novel therapeutic strategies could target key pathobionts or modulate the microbiome to improve patient outcomes, especially in oral health.

### Limitations of this study

This study has several limitations that should be considered. First, the sample size of healthy controls was small, potentially limiting the generalizability of our findings and correlation with clinical findings. Further research with larger control groups is necessary to validate our results and provide more robust conclusions. Second, we were limited to a single cross-sectional time point as longitudinal collections are ongoing.

## MATERIALS AND METHODS

### Participants, collection, and swab samples genomic prep

cwSCD who were <18 years old (*N* = 40) were identified from clinic schedules and recruited during routine surveillance visits at baseline health to participate in this study from Nationwide Children’s Hospital and Children’s Healthcare of Atlanta (IRB STUDY00002085 and IRB STUDY00005127). Exclusion criteria for cwSCD included systemic corticosteroid or acute antibiotic usage in the month prior to enrollment (excluding prophylactic antibiotics), transplantation history, chronic blood transfusions, or other immunodeficiencies besides functional or surgical asplenia. Recruitment of patients with acute respiratory symptoms was deferred until symptom resolution. Available siblings of participants who were <18 years old and did not have SCD were recruited as the healthy control population (*N* = 8). Nasopharyngeal and oropharyngeal swab samples were collected using OMNIgene ORAL kits (DNAgenotek, OMR-110). Each nasal and oral swab was extracted for DNA using the Qiagen QIAamp DNA microbiome kit according to the manufacturer’s instructions, which includes a human DNA removal step that allows for its use in low microbial biomass niches. DNA was quantified using a Qubit 4.0 fluorimeter and measured for purity with a Nanodrop One spectrophotometer.

### Sequencing library generation and processing

Sequencing libraries were constructed using the NEBNext Ultra II FS DNA Library Prep Kit according to the manufacturer’s instructions. Libraries were quantified using a Qubit 4.0 Fluorimeter and checked for fragment distributions with an Agilent 2100 Bioanalyzer using a High Sensitivity DNA Kit. A no DNA control verified that there was no contaminating DNA in our sequencing libraries. Libraries were pooled in equimolar ratios and sequenced on an Illumina NovaSeq 6000 as 150 bp paired-end reads at a depth of 40 million reads per sample.

The quality of sequencing libraries was assessed with FastQC v0.11.7, and reads were trimmed with Trimmomatic v0.36 (61). Human reads were filtered out using BBDuk with the hg38 human reference genome (62). Samples were verified to have human gDNA removed by performing bowtie2 alignments (63).

### Taxonomical classification of reads

The remaining reads from quality control processing were merged with PANDAseq v2.11 and taxonomically assigned using Kraken2 v2.1.2 (64, 65). For this manuscript, a custom Kraken2 database was built using all available genomes from NCBI Refseq, encompassing taxonomic data from 320,416 bacteria, 1,857 archaea, 550 fungi, and 96 protozoan species and strains (accessed on 6 June 2023). Low-complexity sequences were masked during the build of this database.

### Figures and statistics

Statistical tests were performed in GraphPad Prism 10 (descriptive statistics, Mann-Whitney). PERMANOVAs were performed in R-Studio. Alpha diversity Hill metric was performed in R-Studio. Beta diversity metrics Bray-Curtis ordinations were performed in R-Studio. LEfSe analysis was performed using lefse v1.1.2 on command line (29).

## Data Availability

All metagenomic sequencing information has been uploaded to the Sequence Read Archive (SRA) under BioProject accession ID PRJNA1222334.

## SUPPLEMENTAL MATERIAL

Supplemental figures msphere.00137-25-s0001.pdf Figures S1–S5.

ASM does not own the copyrights to Supplemental Material that may be linked to, or accessed through, an article. The authors have granted ASM a non-exclusive, world-wide license to publish the Supplemental Material files. Please contact the corresponding author directly for reuse.
