# Supplementary material for: Altered nasal and oral microbiomes define pediatric sickle cell disease
Source: mSphere. 2025 May 14;10(6):e00137-25. doi: 10.1128/msphere.00137-25 (PMC12188711; doi:10.1128/msphere.00137-25)
Supplement: Supplemental figures — Figures S1–S5. [file msphere.00137-25-s0001.pdf]

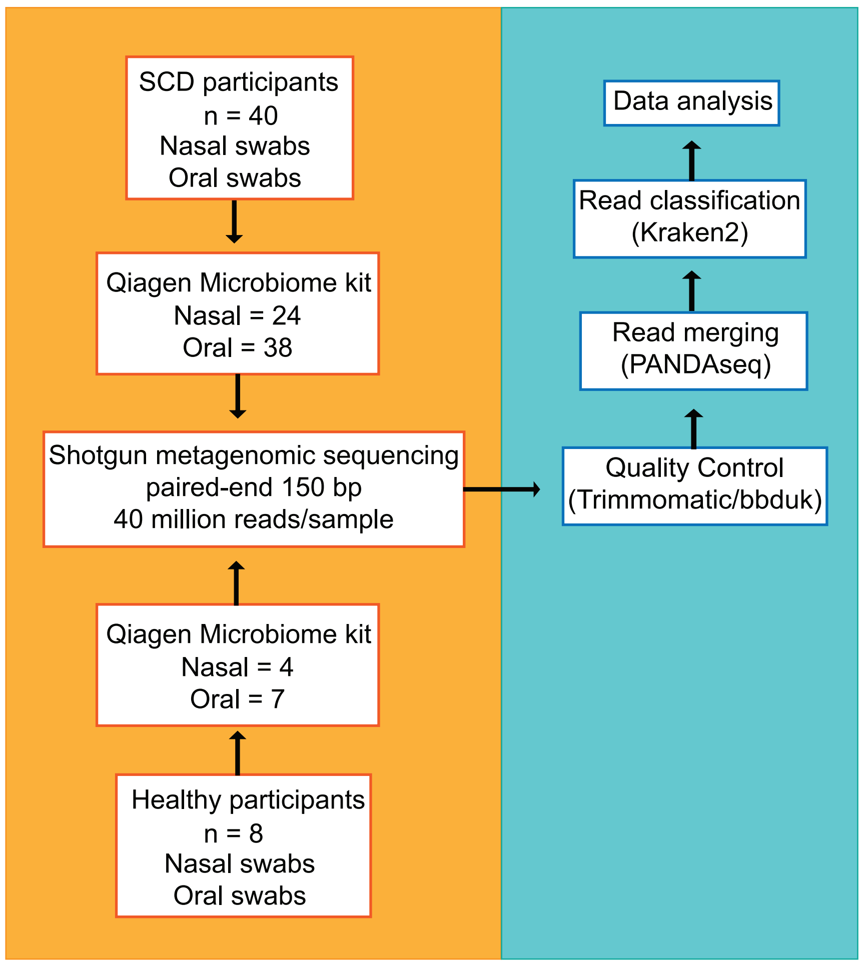

Supplemental Figure 1. Sample and bioinformatic processing map.

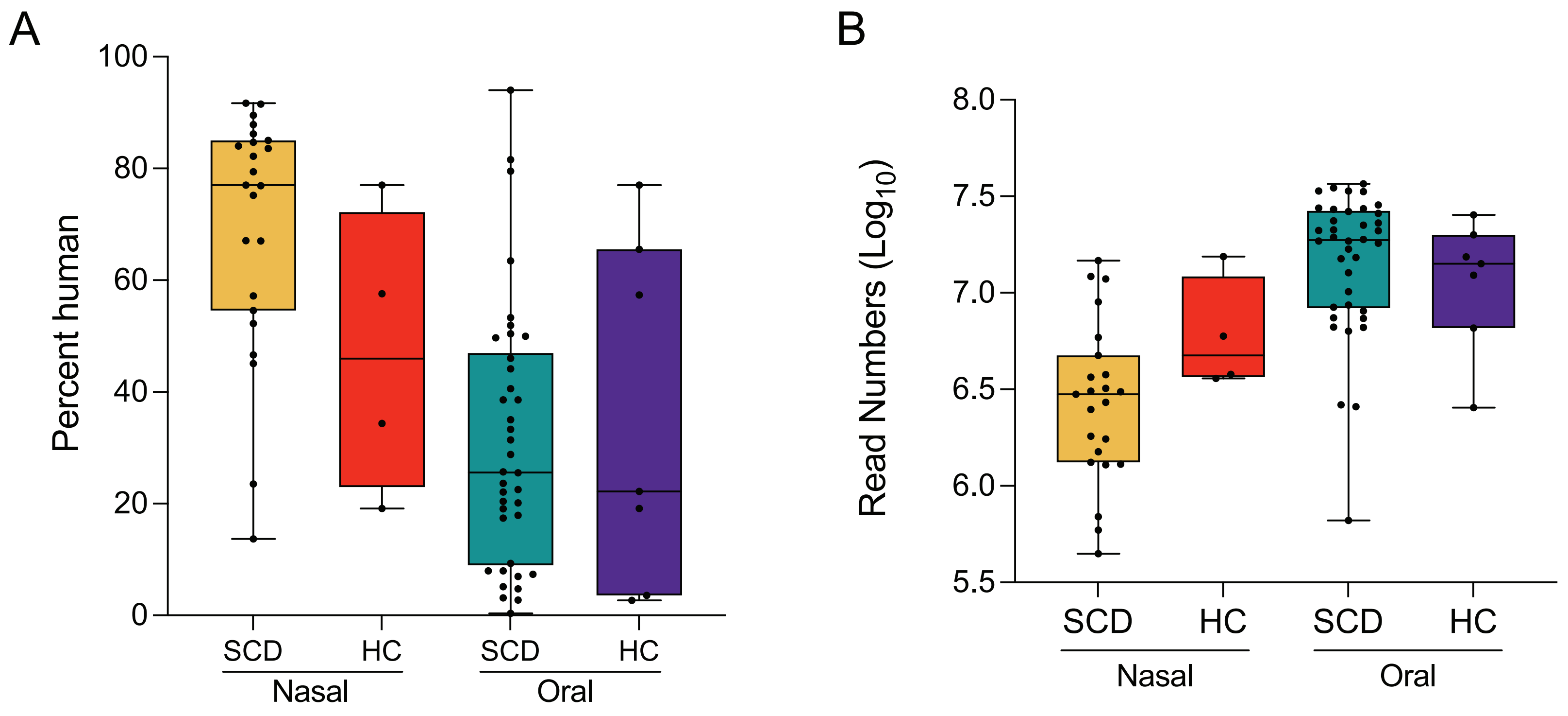

Supplemental Figure 2. Sequencing library features for the oral and nasal microbiome samples. A. The level of human contamination found for each microbiome sample for cwSCD and HC individuals. **B.** The log10 value of reads for each microbiome sample following depletion of human DNA and sequencing is plotted for cwSCD and HC individuals. Nasal samples: N = 23 for SCD, N = 4 for HC. Oral samples: N = 38 for cwSCD, N = 7 for HC. Goldenrod represents nasal cwSCD samples, and red represents nasal HC samples. Teal represents oral cwSCD samples, and purple represents oral HC samples.

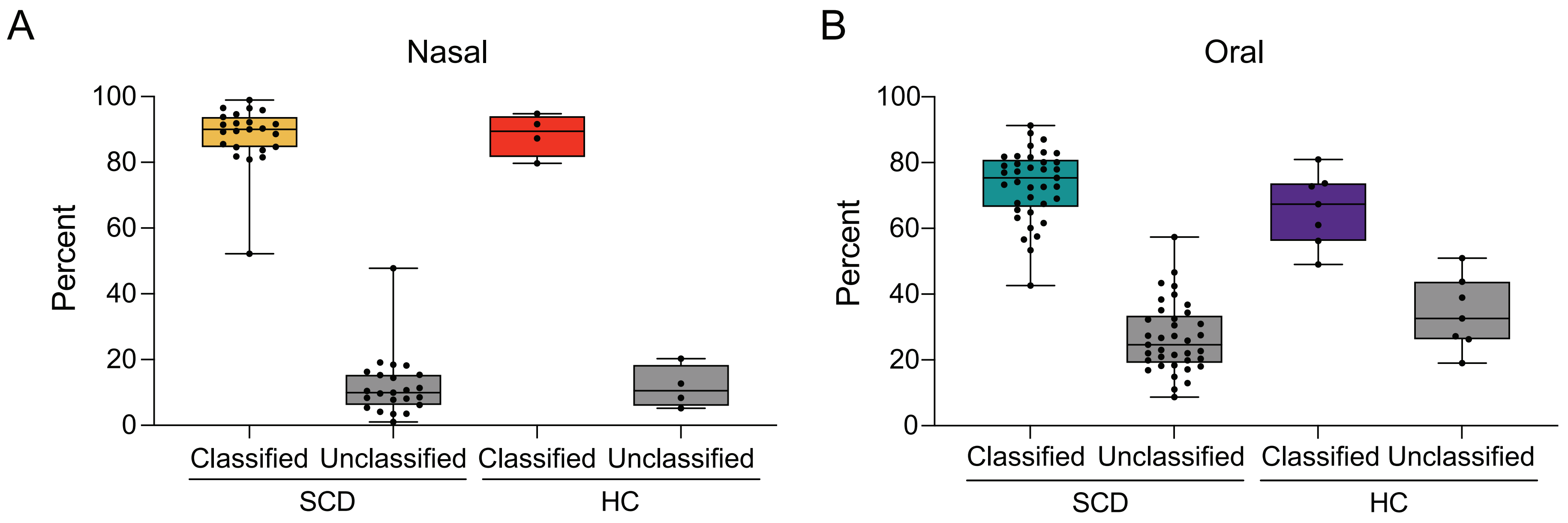

Supplemental Figure 3. Unclassified reads are similar between cwSCD and HC subjects. Reads from each microbiome samples were taxonomically assigned using Kraken2. The percentage of reads in assigned and unassigned bins was plotted for each cwSCD and HC sample for the nasal (**A**) or oral (**B**) cavity. Nasal samples: N = 23 for cwSCD, N = 4 for HC. Oral samples: N = 38 for cwSCD, N = 7 for HC. Goldenrod represents nasal cwSCD samples, and red represents nasal HC samples. Teal represents oral cwSCD samples, and purple represents oral HC samples.

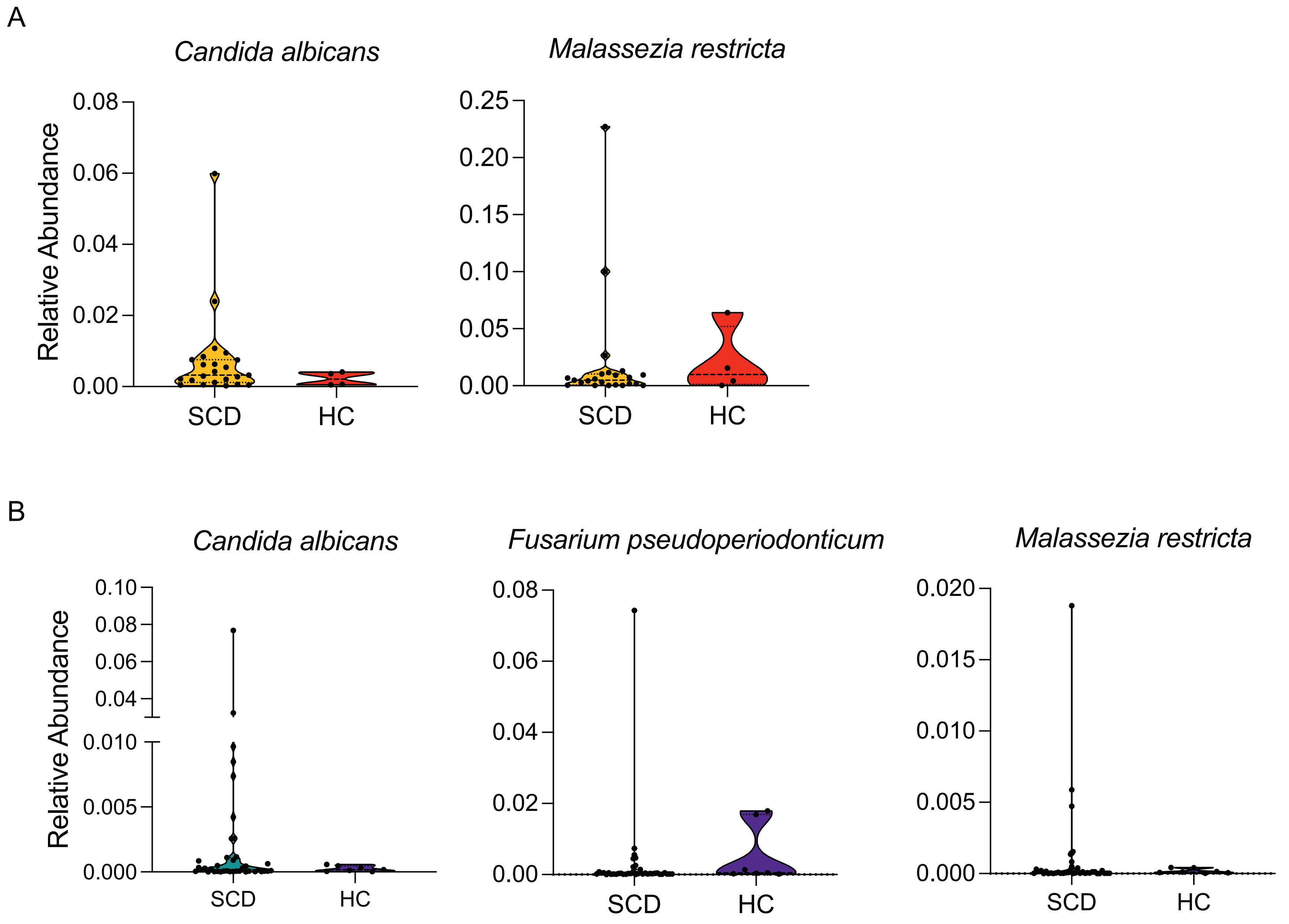

Supplemental Figure 4: Abundant fungal taxa were similar in cwSCD and healthy control individuals. The relative abundance of fungal species detected in the nasal (**A**) or oral (**B**) cavity at greater than 5% in one or more samples. Nasal samples: N = 23 for cwSCD, N = 4 for HC Oral samples: N = 38 for cwSCD, N = 7 for HC. Goldenrod represents cwSCD samples, and red represents HC samples. Teal represents oral cwSCD samples, and purple represents oral HC samples.

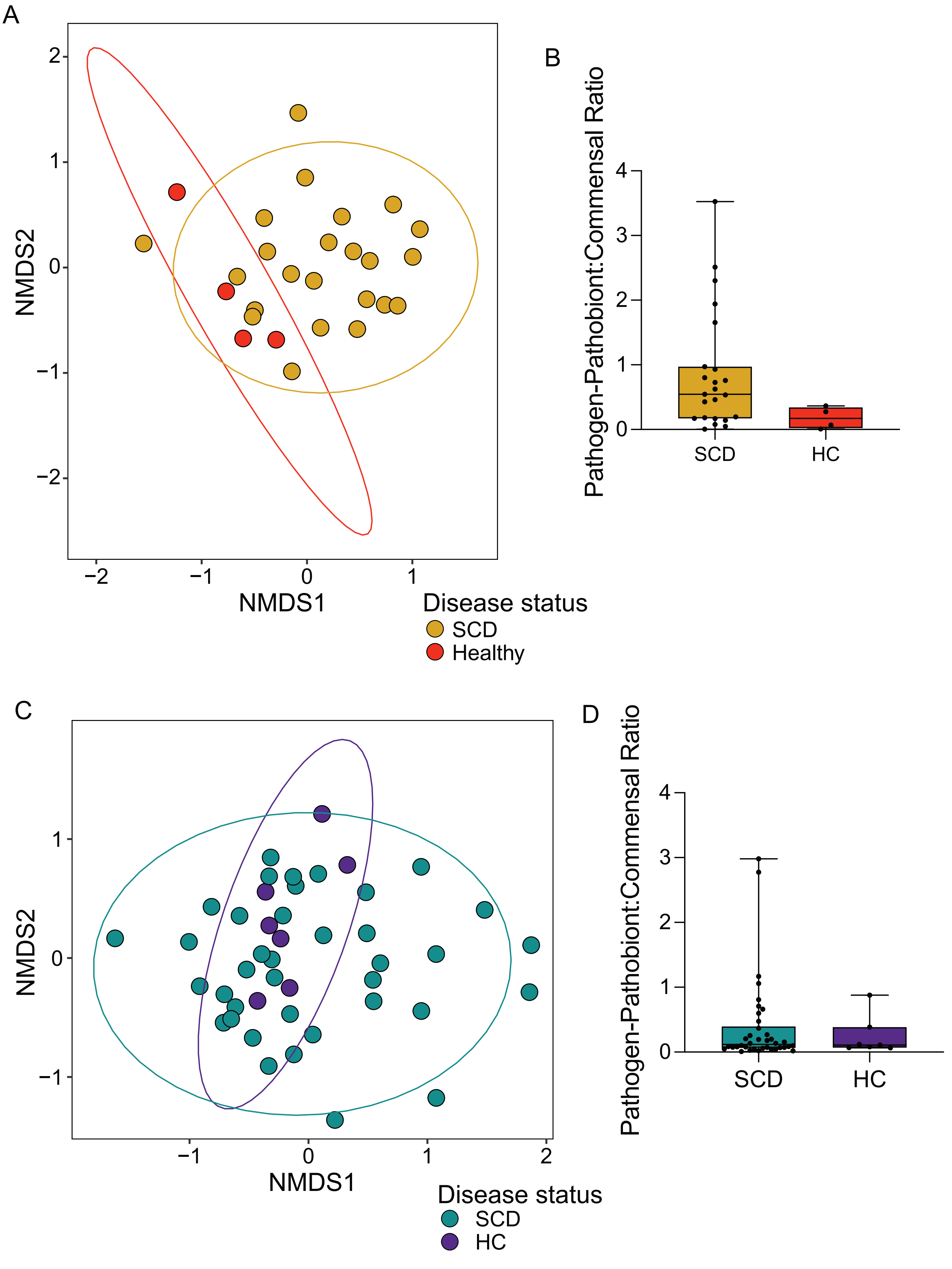

Supplemental Figure 5: cwSCD microbiome samples do not contain greater amounts of pathobionts or pathogens than HC individuals. The abundance of pathogen and pathobiont species in nasal (**A**) and oral (**C**) samples were visualized using Bray-Curtis dissimilarity index for β-diversity for cwSCD and healthy control subjects. Calculated ratio of pathogen-pathobiont:commensal species for nasal (**B**) and oral (**D**) samples. Nasal: N = 38 for cwSCD, N = 7 for HC. Oral: N = 38 for cwSCD, N = 7 for HC. Goldenrod represents cwSCD samples, and red represents HC samples. Teal represents oral cwSCD samples, and purple represents oral HC samples.
